# Er:YAG Laser Assisted Treatment of Central Odontogenic Fibroma of the Mandible

**DOI:** 10.1155/2015/230297

**Published:** 2015-09-20

**Authors:** Luis Silva Monteiro, Marco Martins, José Júlio Pacheco, Filomena Salazar, João Magalhães, Paolo Vescovi, Marco Meleti

**Affiliations:** ^1^Department of Medicine and Oral Surgery, Higher Institute of Health Sciences (ISCS-N), CESPU, 4585-116 Paredes, Portugal; ^2^Institute of Research and Advanced Training in Health Sciences and Technologies (IINFACTS), Higher Institute of Health Sciences (ISCS-N), CESPU, 4585-116 Paredes, Portugal; ^3^Stomatology and Dental Medicine Department, Centro Hospitalar de São João, Polo de Valongo, 4440-563 Valongo, Portugal; ^4^Department of Biomedical, Biotechnological and Translational Sciences, Center of Oral Laser Surgery and Oral Pathology, Dental School, University of Parma, 43125 Parma, Italy

## Abstract

Central odontogenic fibroma is a very rare benign odontogenic tumour characterized by a fibrous mature stroma with variable strands or islands of inactive-looking odontogenic epithelium. Our aim is to report a case of a central odontogenic fibroma and describe the clinical usefulness of Er:YAG laser for the surgical treatment of this tumour. A 74-year-old woman presented with an expansive lesion located in a mandible with multilocular and mixed radiographic appearance. A conservative excision using Er:YAG laser was performed. Complete removal was obtained. There were no postoperative complications. The histopatologic features were consistent with the diagnosis of central odontogenic fibroma of rich-epithelium type. No recurrence was observed during follow-up.

## 1. Introduction

Central odontogenic fibroma (COF) is a very rare benign neoplasm characterized by a fibrous mature stroma with variable strands or islands of inactive-looking odontogenic epithelium [1]. COF accounts for less than 0.1% of oral lesions and 1.5% of all odontogenic tumours [2, 3]. They appear in a wide age group (4–80 years old) with predilection for females [4]. Despite the initial report of mandible predilection [5], more recent data describes an equal frequency of this lesion on the mandible and the maxilla [6, 7]. In the maxilla the tumour appears to frequently involve the anterior region, whereas in the mandible it involves more frequently the posterior region, with most lesions located in the premolar or molar area [7–9].

Clinically, COF may appear as an asymptomatic slow growing lesion associated with cortical expansion. Radiographically COF presents as a unilocular or multilocular radiolucent area or sometimes with a mixed radiolucent/radiopaque appearance [4]. Bilateral COF had been reported [10]. Root resorption and teeth displacement have been reported in cases of more severe lesions [11–13]. COF responds well to conservative surgical exeresis with no tendency to recur or to undergo malignant transformation [4, 9].

Here we report a case of a central odontogenic fibroma of the mandible with partially ill-defined margin treated with conservative excision using Er:YAG laser. The clinical, imaging, surgical, and pathological findings are discussed altogether with a brief review of the literature.

## 2. Clinical Case

A 74-year-old Caucasian Portuguese woman was referred to our Oral Medicine and Surgery Department by her general practitioner because of a painless swelling of the mandible. The patient reported that the nodular lesion was present for 8 years without pain and notable changes in size. Anamnesis disclosed presence of osteoporosis, hypercholesterolemia, arterial hypertension, and peripheral venous insufficiency.

**Figure 1:**
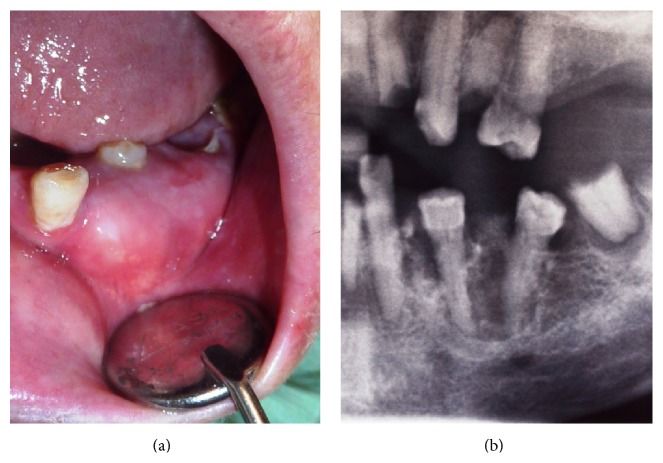
(a) Intraoral view of a left mandible enlargement with displacement and submersion of involved teeth. (b) Panoramic radiographic image (partial view) showing a multilocular radiolucent lesion with calcified foci.

At oral examination an enlargement of the mandible in buccal left premolar region extending from the canine to the first molar (Figure 1(a)) was evident. The overlaying mucosa was of normal colour and texture. This nodule of 2 × 2 cm was firm and painless and had a nontender bony-hard consistency with no fluctuation on manual palpation. Both premolars were vital, and the second one had a partial crown fracture. Both were covered by the lesion. Root remnants of first and second molars were present. The patient had other radicular fragments, dental caries, and periapical periodontitis. No associated cervical lymphadenopathy was detected.

**Figure 2:**
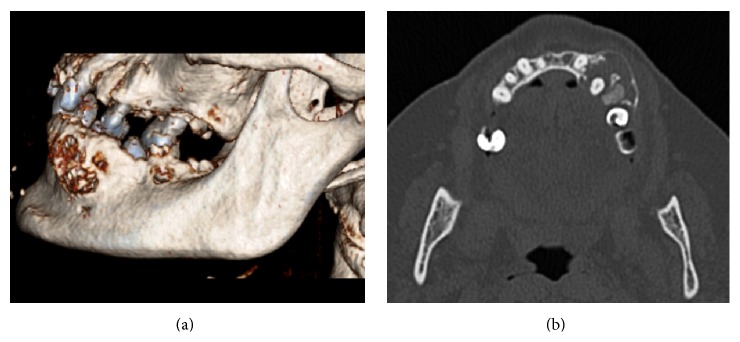
(a) Three-dimensional computed tomography (CT) reconstruction showing an expansion of the left-side of the mandible with some erosion areas on buccal cortical surface. (b) Axial CT image showing a low-density multilocular lesion.

Panoramic radiography showed a radiolucent lesion with a multilocular appearance between the mandibular left canine and second premolar (Figure 1(b)). The upper margin of the lesion was well-defined, contrary to the ill-defined lower margin. Root resorption was absent. CT scan revealed a multilocular lesion containing small calcified masses (Figure 2). This lesion, sized 2.2 × 1.5 × 2.1 cm, involved and displaced 34 and 35 teeth, with expansion and thinning of the buccal and lingual cortical plates partially eroded. The inferior margin of the lesion showed a proximity relation with mandibular canal and mental foramen.

Differential diagnosis included odontogenic myxoma, central giant cell granuloma, osseous dysplasia, desmoplastic fibroma, calcifying epithelial odontogenic tumour, keratocystic odontogenic tumour, ameloblastic fibroma, and ameloblastoma.

**Figure 3:**
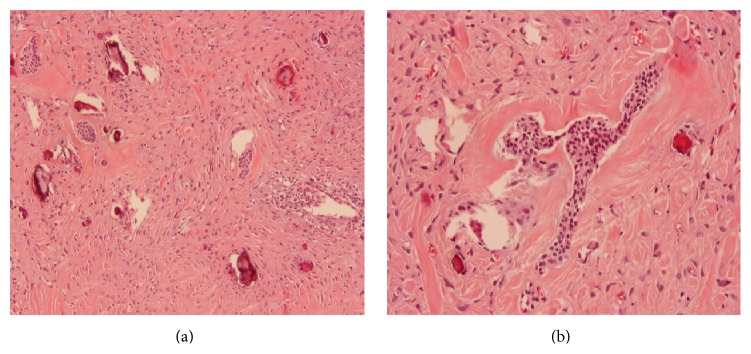
(a) Microscopic view of the lesion demonstrating the presence of fibroblastic and collagenous tissue with many strands of odontogenic epithelial cells and focally calcified foci resembling osteoid and cementoid material (H&E stain ×100 magnification). (b) Odontogenic epithelial nests without cytological atypia in a fibroblastic and collagenous stroma with hyaline areas (H&E stain ×200 magnification).

**Figure 4:**
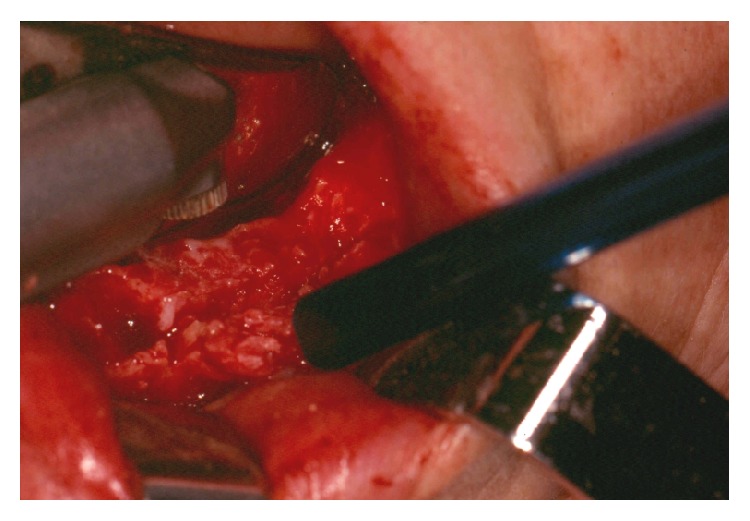
Excision of the tumour performed with Er:YAG laser (2940 nm).

**Figure 5:**
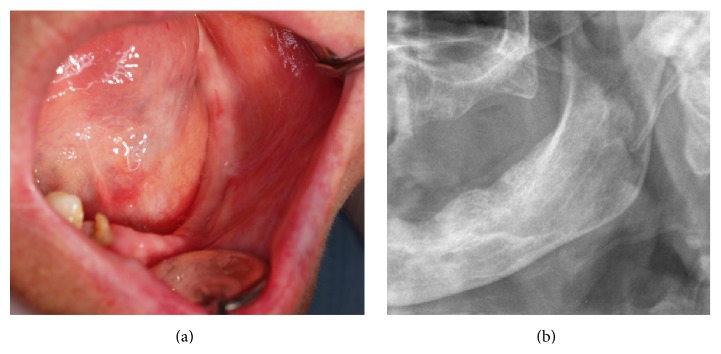
Clinical and radiographic appearance one year after excision of the lesion without signs of recurrence ((a) intraoral view and (b) panoramic radiographic image).

Extraction of 37, 36, 35, and 34 was performed and a bioptic specimen of the lesion was obtained under local anaesthesia (2% lidocaine with 1 : 100,000 epinephrine). Histopathological evaluation showed a lesion composed of a fibroblastic/collagenous tissue with many strands or nests of odontogenic epithelial cells without cytological atypia. Focally prominent hyaline calcified foci, resembling osteoid and dentinoid material, were also present (Figure 3). Such features favoured the diagnosis of a central odontogenic fibroma. After one month, the patient underwent the excision of the lesion under local anaesthesia using Er:YAG (2940 nm) laser for bone osteotomy (Figure 4). We used a Deka Smart 2940 Plus laser, with articulated arm, with an angulated mirror hand piece, 1 mm of spot, on a short-pulse mode, 15 Hz, 300 mJ, 4.5 W output power, power density of 573.25 W/cm^2^, and fluence of 38.22 J/cm^2^. During the excision, we observed that the lesion was almost encapsulated except at its inferior margin with a more ill-defined border. Because of this, additionally to the enucleation and curettage of the lesion, a peripheral osteotomy was performed until clear bone tissue was obtained to ensure complete elimination of the tumour. There were no postoperative complications such as pain, paresthesia, and swelling one week after surgery. Histopathologic examination of the whole specimen confirmed the diagnosis of central odontogenic fibroma, epithelium-rich type (complex or WHO type). No recurrence was seen at the follow-up visits (Figure 5) during a period of 4 years.

## 3. Discussion

Central odontogenic fibroma is a very rare benign odontogenic tumour, described in less than a hundred cases in indexed literature [3, 4, 9, 10, 14–17]. Initial descriptions were somewhat confusing, with many central fibrous lesions such as hyperplastic dental follicle, desmoplastic fibroma, or myxofibroma classified as an odontogenic fibroma [5, 18]. In 2005, the World Health Organization (WHO) identified two separate histological types of central odontogenic fibroma: the epithelium-poor type (formerly termed simple type) and the epithelium-rich type (formerly termed complex or WHO-type). The first is a noninfiltrating connective tissue lesion resembling a dental follicle, minimally cellular with dispersed delicate collagen fibres. Scattered remnants of inactive-looking odontogenic epithelium appear as small irregular islands and cords. Foci of dystrophic calcifications may be present. The epithelium-rich type COF is composed of a more cellular fibroblastic connective tissue interwoven with less cellular and often vascular areas. Islands or strands of inactive-looking odontogenic epithelium are an integral component. In this type, typical foci of calcified material are present and considered to be dysplastic cementum, osteoid tissue, or dentin [1]. It has been speculated that this type of COF arises from periodontal ligament [18]. Indeed, many of the cases simulate periodontal diseases [19, 20]. Some tumours may have additional features like pleomorphic fibroblasts, granular cells, giant cells, or desmoplastic stroma [13, 21, 22]. On the basis of the presence of fibroblastic/collagenous tissue with many strands or nests of odontogenic epithelial cells and calcified foci resembling osteoid and dentinoid material, the present case has been diagnosed as epithelium-rich type (formerly termed complex or WHO-type).

The clinical characteristics of COFs observed in our case are within the reported in the literature [4, 9]. The radiographic features of this tumour are not pathognomonic [23]. Although in the majority of cases they are radiolucent and unilocular with well-defined borders, there are many reported cases with multilocular appearance, with scalloped margins and mixed image [4, 23]. Daniels [4] suggested that smaller lesions are unilocular while larger ones tend to have scalloped margins or are multilocular. In the present case we observed a multilocular radiolucent lesion with calcifications foci both on panoramic radiography and in the CT scan. Radiographic differential diagnosis includes odontogenic myxoma, central ossifying fibroma, central giant cell granuloma, some types of fibrous dysplasia, traumatic bone cyst, osseous dysplasia, calcifying epithelial odontogenic tumour, keratocystic odontogenic tumour, ameloblastoma, and ameloblastic fibroma [9, 24–26]. Definitive diagnosis lays on histological examination. Differentiation of these lesions is very important since some lesions including ameloblastic fibroma, odontogenic myxoma, or desmoplastic fibroma are more aggressive than COF [9, 26]. On histological examination, odontogenic myxoma is composed of rich myxoid or mucoid stroma with few collagen fibrils and has few odontogenic epithelium islands or it may be absent in many cases. Desmoplastic fibroma presents interlaced bundles of densely collagenous tissue containing uniform spindled shaped cells. Ameloblastic fibroma consists in an anastomosing proliferative odontogenic epithelium with peripheral rim of columnar cells in a primitive connective tissue stroma [26]. Microscopically, our case had a fibrocollagenous stroma without myxoid pattern and had many epithelial islands that resemble odontogenic epithelium but not ameloblastic cells. These characteristics discard odontogenic myxoma, desmoplastic fibroma, or ameloblastic fibroma. Additionally, we found calcified material similar to dysplastic cementum, osteoid, or dentin. This is a characteristic of central odontogenic fibroma of rich-epithelium type.

Conservative excision through enucleation and curettage of the lesion is the treatment of choice for COF [4, 9, 17]. Because of the ill-defined inferior margin and proximity relation of the lesion with the mental foramen, we performed a peripheral osteotomy with a Er:YAG laser, with an noncontact technique allowing an excellent visualization of bone removal without any instrument as a bur or chisel over the operating hard tissue. This allowed us to have an immediate and clear visualization of the progressive elimination of bone tissue respecting anatomical structures. The neurovascular involvement is reported in central odontogenic fibroma [27]. Er:YAG laser is a solid-state laser where the active medium is a crystal of yttrium-aluminium-garnet doped with erbium. This laser has a wavelength of 2940 nm and produces excellent absorption of hydroxyapatite and water. The osteotomy performed with this laser has proved to be efficient and provides clean and high precision cut with minimal injury to the adjacent hard and soft tissue. It has also the advantage of inducing a lower increase in temperature of bone with respect to conventional burs. Other advantages include bactericidal and biostimulatory properties, the possibility of faster and greater bone regeneration, and less painful healing compared to traditional surgery [28–32]. The cost of the laser equipment could be, however, a limitation for its use. This laser has been used in the treatment of several bone pathologies including odontogenic tumours [28, 29, 33]. Angiero et al. [33] reported clear advantages using a surgery laser protocol compared with conventional surgery in a randomized controlled study of patients with odontomas. Nevertheless, to the best of our knowledge, the case presented here is the first report of a central odontogenic fibroma treated surgically using Er:YAG laser.

Recurrence is uncommon [4, 26, 34]. Some reported cases with recurrence were associated with incomplete surgical removal of the lesion and sometimes due to nonencapsulated margins [7, 35]. In the present case, we think that Er:YAG laser was a useful instrument for the elimination of the entire lesion especially in ill-defined areas. The follow-up showed good bone healing and no signs of recurrence.

In conclusion, the radiological and histological features of the case reported here were consistent with the diagnosis of central odontogenic fibroma of rich-epithelium. The conservative excision using Er:YAG laser appears to be an effective procedure for the treatment of this very rare odontogenic tumour.
